# Supplementary material for: Systematic analysis of bypass suppression of essential genes
Source: Mol Syst Biol. 2020 Sep 17;16(9):e9828. doi: 10.15252/msb.20209828 (PMC7507402; doi:10.15252/msb.20209828)
Supplement: Supplementary file 1 — Appendix [file MSB-16-e9828-s001.pdf]

Appendix for

Systematic analysis of bypass suppression of essential genes

Jolanda van Leeuwen, Carles Pons, Guihong Tan, Jason Zi Wang, Jing Hou, Jochen Weile, Marinella Gebbia, Wendy Liang, Ermira Shuteriqi, Zhijian Li, Maykel Lopes, Matej Ušaj, Andreia Dos Santos Lopes, Natascha van Lieshout, Chad L. Myers, Frederick P. Roth, Patrick Aloy, Brenda J. Andrews, and Charles Boone

Correspondence to: jolanda.vanleeuwen@unil.ch (J.v.L.); charlie.boone@utoronto.ca (C.B.); brenda.andrews@utoronto.ca (B.J.A.);

Table of Contents

Item	Page
Appendix Figure S1. Suppressor isolation, identification and confirmation	2
Appendix Figure S2. Mechanisms of suppression	3
Appendix Figure S3. Suppression by aneuploidies	4
Appendix Figure S4. Partial aneuploidies and suppressor prediction	5
Appendix Figure S5. Evolutionary properties of dispensable essential genes	6
Appendix Figure S6. Properties of dispensable essential human genes	7
Appendix Figure S7. Dispensable essential gene prediction	9
Appendix Figure S8. Bypass suppressors identified in *S. Pombe*	10

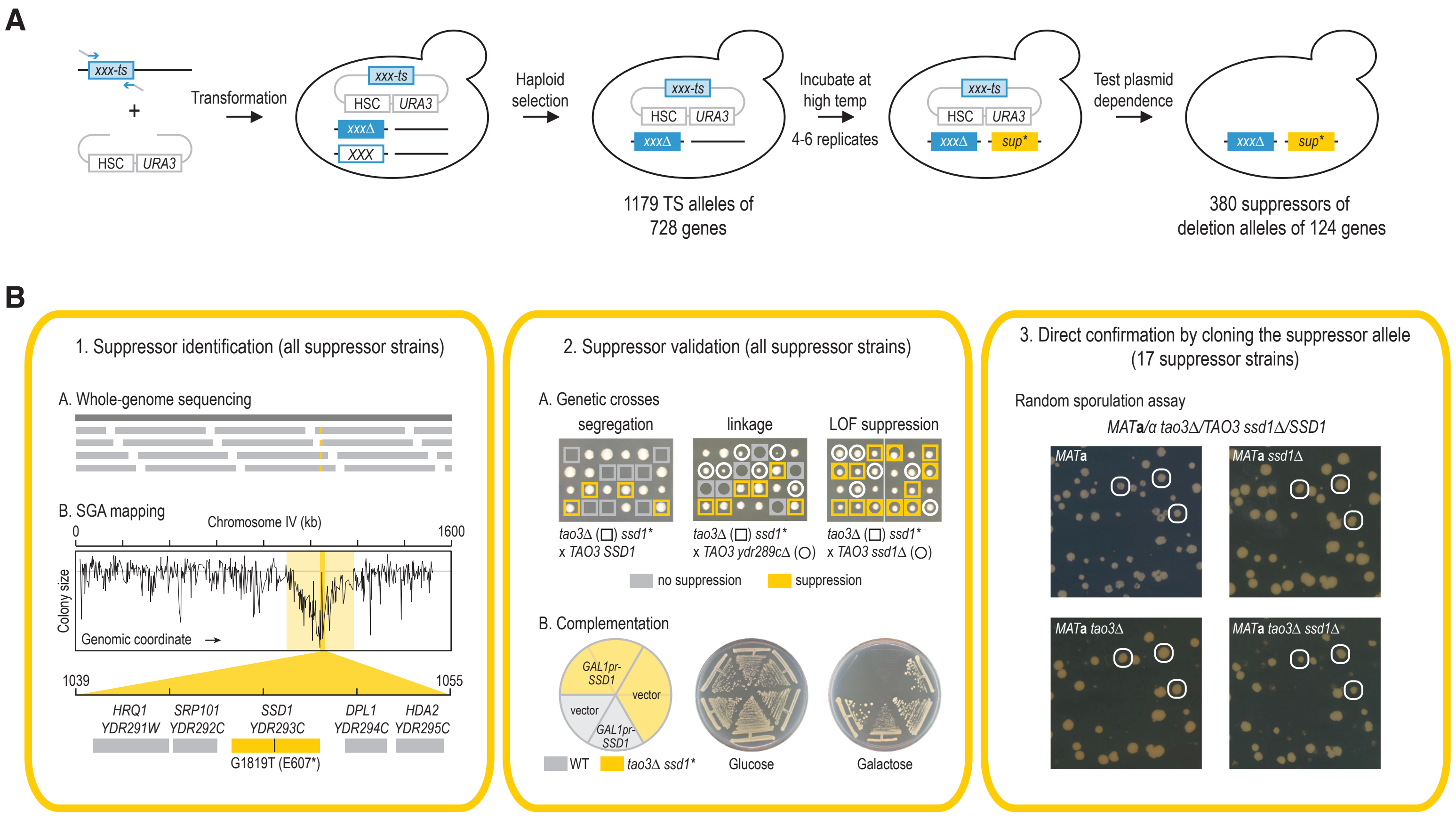

Fig. S1. Suppressor isolation, identification and confirmation.

A Strategy for isolating bypass suppressors of essential gene deletion mutant alleles. 1179 temperature sensitive (TS) alleles were amplified from available TS strains, thereby including regions of homology to a plasmid carrying the counter-selectable marker *URA3* and a haploid-selection cassette (HSC). The PCR product and linearized plasmid were cotransformed into a diploid yeast strain that was heterozygous for a deletion allele of the corresponding essential gene. The resulting diploid strains carrying an assembled plasmid were sporulated, and haploid progeny carrying the deletion allele of the essential gene and the TS allele on plasmid were selected using the haploid selection cassette present on the plasmid. For each strain, ~25 million cells were subsequently incubated at a restrictive temperature for 4 days, and suppressor colonies were isolated and tested for growth on media containing 5-FOA, which selects for loss of the plasmid. Suppressor isolation was repeated 4-6 times for each strain. In total, 380 bypass suppressor strains were isolated, for 124 essential gene deletion mutants.

B Suppressor identification and validation. 1) All 380 suppressor strains were sequenced whole-genome to identify the suppressor mutations. To complement the sequencing data, 89 haploid suppressor strains that had a relatively mild fitness defect were screened by Synthetic Genetic Array (SGA) analysis to identify the genomic location of the suppressor mutation as a stretch of slow-growing colonies corresponding to mutants that have a genomic location in close proximity to the suppressor mutation (Jorgensen et al., 2002). SGA data of a *tao3Δ* mutant that is suppressed by a nonsense mutation in *SSD1* is shown as an example. 2) Suppression interactions were validated using several assays. First, the query strain carrying the suppressor mutation was crossed to a wild-type strain, a strain deleted for a gene linked to the suppressor, and a strain carrying a deletion or conditional allele of the suppressor gene, to determine proper segregation, linkage, and loss-of-function (LOF) suppression, respectively. Next, the query strain carrying the suppressor mutation was transformed with a plasmid carrying the wild-type allele of either the suppressor gene or an empty vector control, and growth was scored. 3) Finally, for 17 suppression interactions, diploid strains were constructed that were heterozygous for both the query and the suppressor mutant alleles. Random sporulation analysis was used to verify the suppression phenotype. In the example that is shown, haploid progeny from a heterozygous *MAT***a***/α tao3Δ/TAO3 ssd1Δ/SSD1* diploid strain were replica plated onto media selecting for the *tao3Δ* allele, the *ssd1Δ* allele, or both. All viable *tao3Δ* colonies also carried a *ssd1Δ* allele, a few example colonies are highlighted. In total, 79% of the tested suppression interactions gave a positive result in at least one of the validation assays. Dataset EV2 contains a summary of the SGA, sequencing, tetrad dissection, complementation, and random sporulation results.

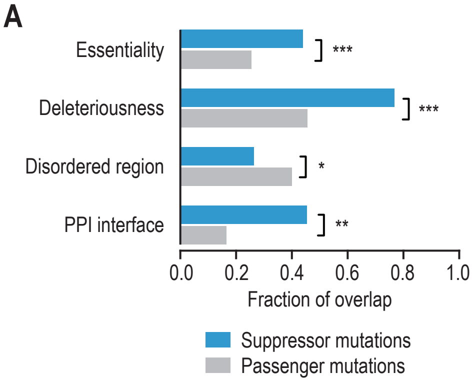

B

Query gene	Paralog gene	Paralog is found as suppressor
MMF1 (YIL051C)	*HMF1 (YER057C)*	No
RPL33A (YPL143W)	*RPL33B (YOR234C)*	No
RPS30B (YOR182C)	*RPS30A (YLR287C-A)*	No
TRR1 (YDR353W)	*TRR2 (YHR106W)*	No
HYP2 (YEL034W)	*ANB1 (YJR047C)*	No
SEC14 (YMR079W)	*SFH1 (YKL091C)*	No
TOR2 (YKL203C)	*TOR1 (YJR066W)*	No
CDC25 (YLR310C)	*SDC25 (YLL016W)*	No
NET1 (YJL076W)	*TOF2 (YKR010C)*	No
NUP116 (YMR047C)	*NUP100 (YKL068W)*	Yes
RPL18A (YOL120C)	*RPL18B (YNL301C)*	Yes
VRG4 (YGL225W)	*HVG1 (YER039C)*	Yes

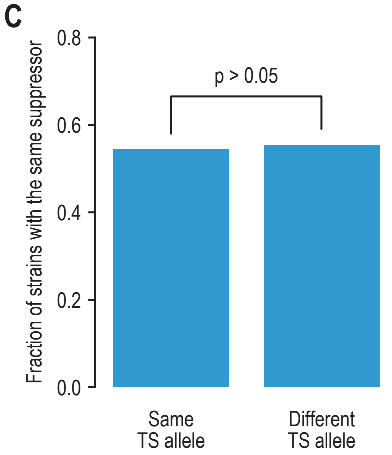

Fig. S2. Mechanisms of suppression.

A The fraction of all suppressor or passenger missense mutations that occur in an essential gene, that are predicted to be deleterious (SIFT score < 0.05), or that map to a disordered region of a protein or to a protein-protein interaction (PPI) interface. * = $p < 0.05$; ** = $p < 0.005$; *** = $p < 0.0005$.

B List of dispensable essential query genes that have a paralog in the yeast genome. The last column indicates whether suppression occurs via mutation of the paralog.

C Fraction of strains with the same suppressor gene for suppressor strains derived from query strains carrying either the same or a different TS allele for the same query gene.

Significance (panels A and C) was determined using Fisher's exact test.

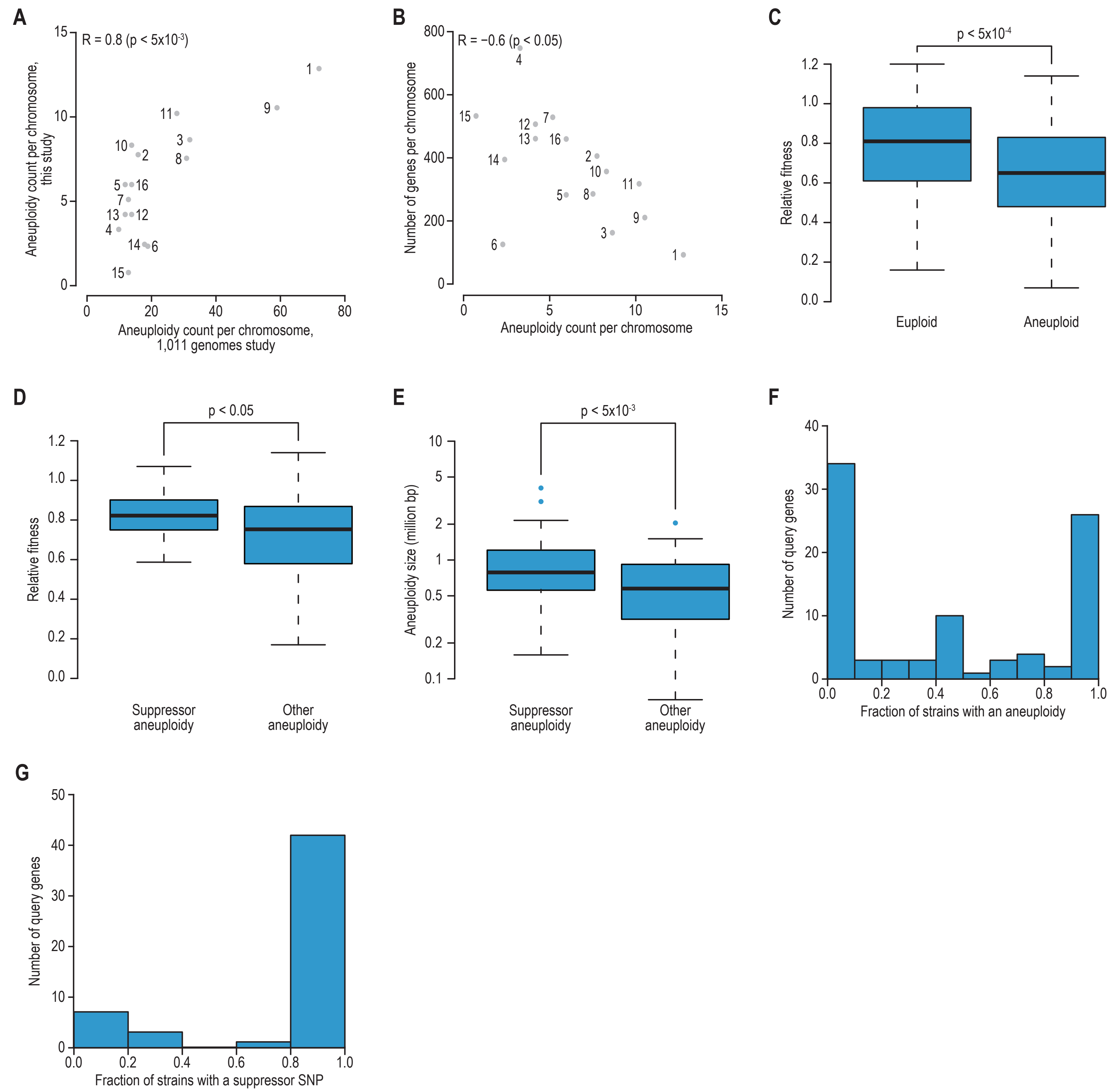

Fig. S3. Suppression by aneuploidies.

A, B The frequency of aneuploidies for each of the 16 yeast chromosomes among the suppressor strains is plotted against (A) the aneuploidy frequency observed in a set of 1,011 natural yeast isolates (Peter *et al*, 2018), and (B) the number of genes on the chromosome. Pearson correlation coefficients and the corresponding p values are indicated. Aneuploidy counts were normalized per query gene.

C, D Fitness distribution of (C) euploid and aneuploid suppressor strains, and (D) aneuploid suppressor strains in which the aneuploidy is either involved in the suppressor phenotype or is a random event not involved in suppression. Statistical significance was calculated using Mann-Whitney's U test.

E Distribution of aneuploidy sizes for aneuploid suppressor strains in which the aneuploidy is either involved in the suppressor phenotype or is a random event not involved in suppression. Significance was determined using Mann-Whitney's U test.

F, G Histograms showing the fraction of suppressor strains for a given query gene that carry an aneuploidy (F) or that are suppressed by SNP (G). Only queries with 2 or more suppressor strains are shown.

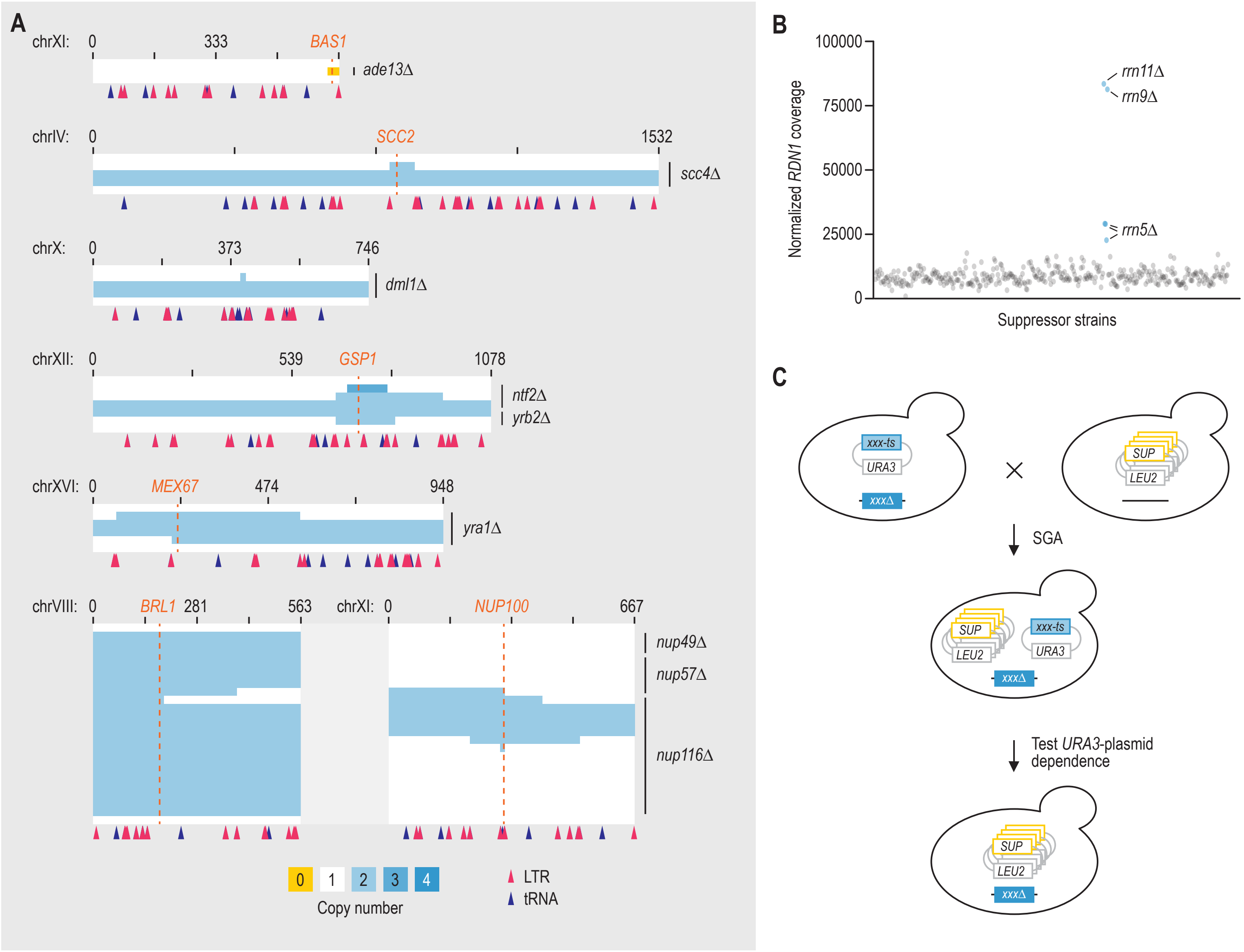

Fig. S4. Partial aneuploidies and suppressor prediction.

A Heatmaps showing genomic copy number based on 30,000 bp regions. Maps are shown for all suppressor isolates of query genes for which at least one of the suppressor isolates carried a partial aneuploidy. Where known, suppressor genes are indicated. LTR = long terminal repeat.

B Normalized *RDN1* coverage is plotted for each of the 380 suppressor strains. Strains with a substantial increase in *RDN1* coverage are highlighted.

C Strategy for identifying causal suppressor genes on aneuploid chromosomes. For each query gene for which suppressor strains carrying aneuploidies were identified, the corresponding parental strain (without the aneuploidy, and with the TS allele on plasmid still present) was crossed into a collection of strains each carrying a multicopy plasmid expressing one of the genes located on the identified aneuploid chromosome. Synthetic genetic array (SGA) analysis was used to isolate haploid progeny carrying the deletion allele of the essential query gene, the corresponding TS allele on plasmid, and the multicopy plasmid. The resulting strains were tested for growth in the absence of the plasmid carrying the TS allele, to determine whether overexpression of the gene located on the multicopy plasmid could rescue the lethality caused by deletion of the essential query gene.

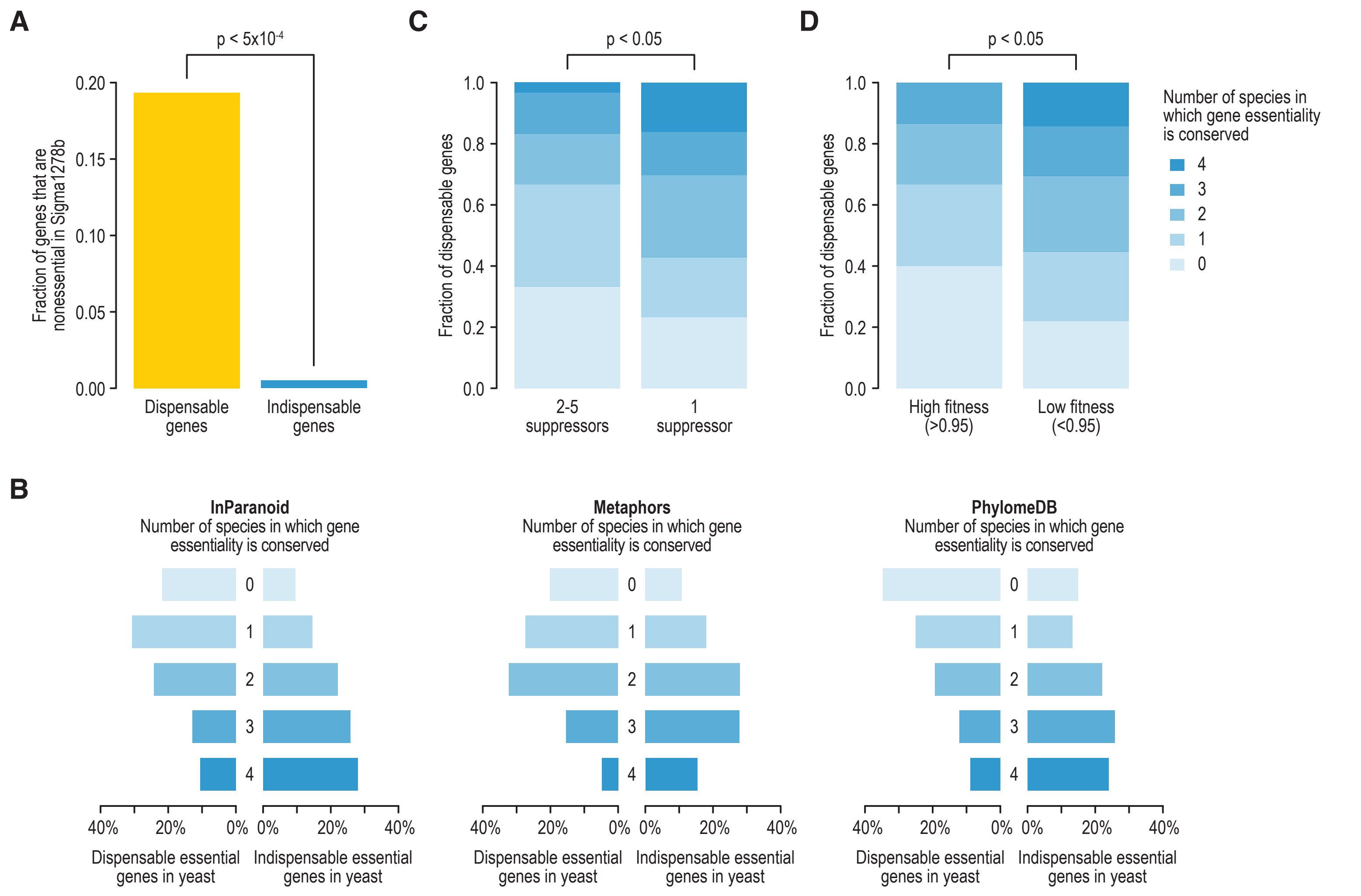

Fig. S5. Evolutionary properties of dispensable essential genes.

A Fraction of dispensable and indispensable essential query genes in S288c that are nonessential in *Saccharomyces cerevisiae* strain Sigma1278b.

B The fraction of dispensable or indispensable essential query genes that are not essential in 0, 1, 2, 3, or 4 of the species *Candida albicans*, *Schizosaccharomyces pombe*, *Caenorhabditis elegans*, and *Homo sapiens* cell lines KBM7/HAP1. Different orthology mapping tools were used to identify orthology relationships.

C, D The fraction of dispensable essential query genes that are not essential in 0, 1, 2, 3, or 4 of the species indicated in (B), split by the number of suppressors that were identified for a query gene (C) or by the average fitness of the obtained suppressor strains for a query gene (D).
All p values were calculated using a 1-sided Fisher's exact test.

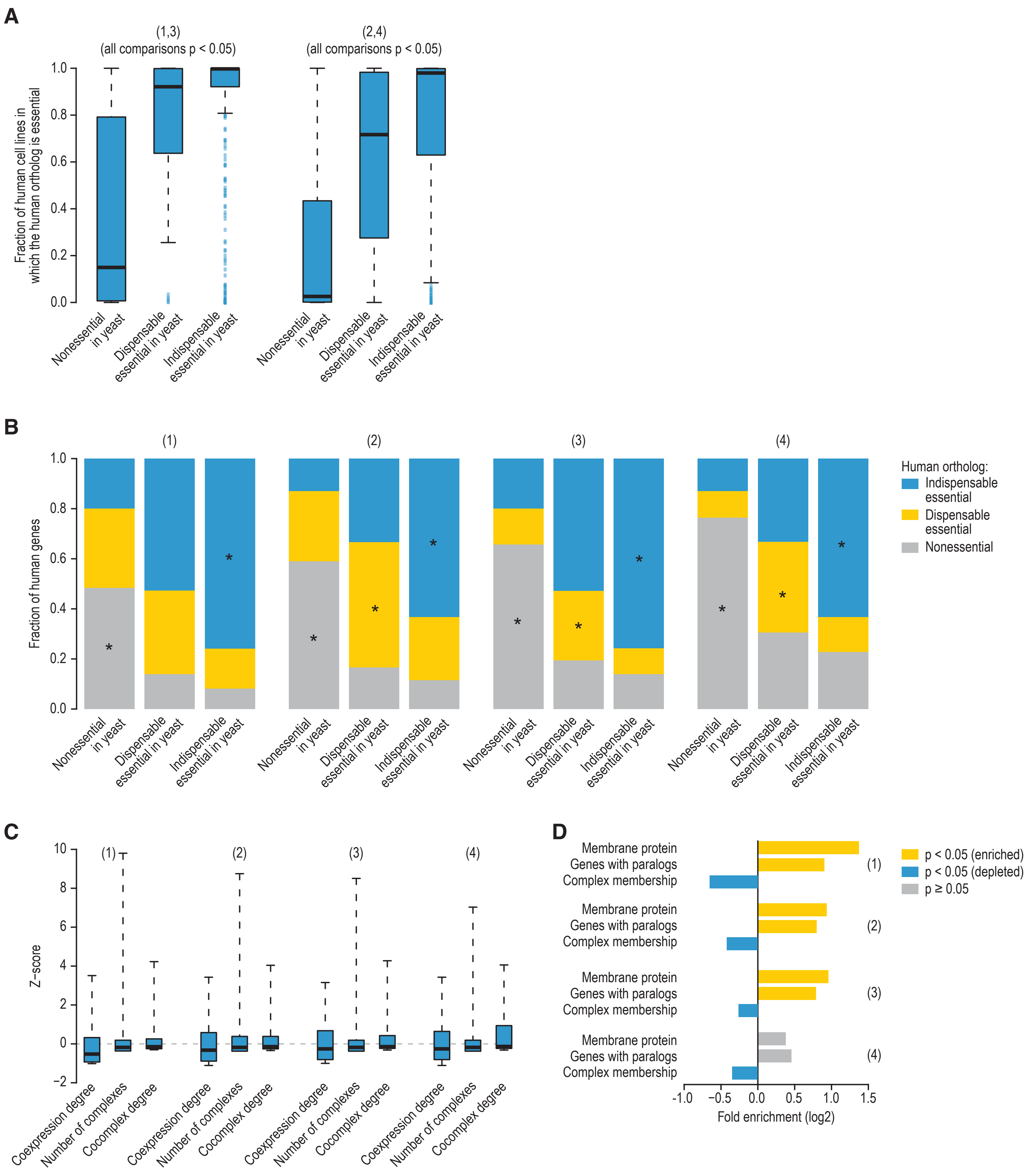

Continued on the next page

E

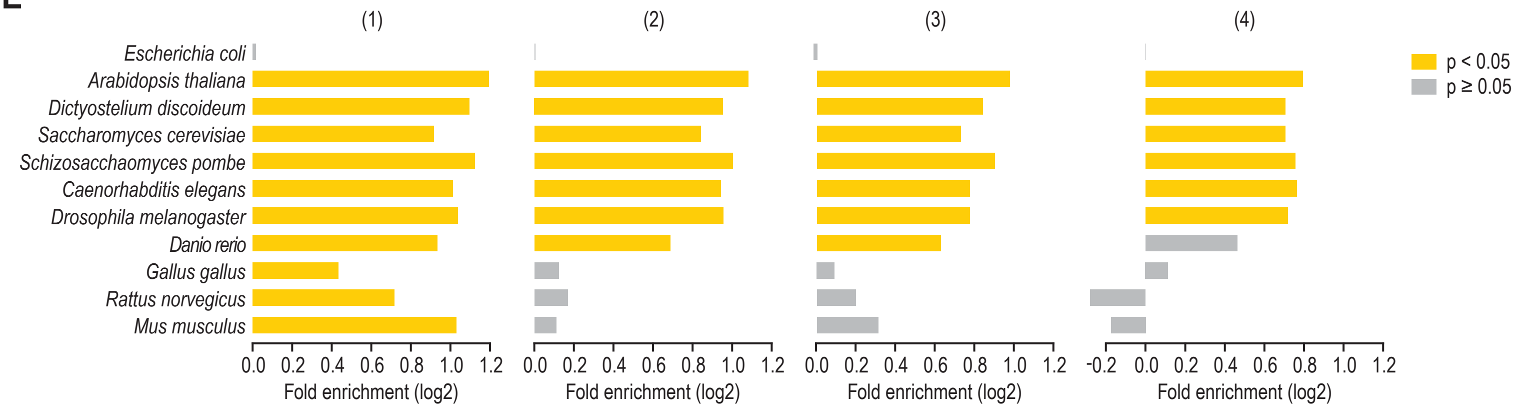

Fig. S6. Properties of dispensable essential human genes.

A Distribution of the fraction of human cell lines in which 1-to-1 orthologs of nonessential, dispensable essential and indispensable essential yeast genes are essential. Statistical significance was determined using a Mann-Whitney's U test.

B Fraction of nonessential, dispensable essential, and indispensable essential query genes in *Saccharomyces cerevisiae* that are nonessential, dispensable essential (= context-depedent or selective essential), or indispensable essential (= core essential) in human cell lines. * = $p < 0.05$ (Fisher's exact test).

C, D Enrichment of dispensable essential human genes for various gene- and protein-level properties. Fisher's exact or Mann-Whitney's U tests were performed to determine statistical significance of the results.

E Fold enrichment for the absence of orthologs of human dispensable essential genes, compared to human indispensable essential genes, in 11 species. P values were calculated using Fisher's exact test.

For all analyses, multiple different thresholds for calling a gene essential in any given cell line or dispensable essential across cell lines were used. (1) essential gene = CERES score < -0.5, dispensable essential gene = essential in 10-90% of cell lines; (2) essential gene = CERES score < -0.7, dispensable essential gene = essential in 10-90% of cell lines; (3) essential gene = CERES score < -0.5, dispensable essential gene = essential in 50-90% of cell lines; (4) essential gene = CERES score < -0.7, dispensable essential gene = essential in 50-90% of cell lines.

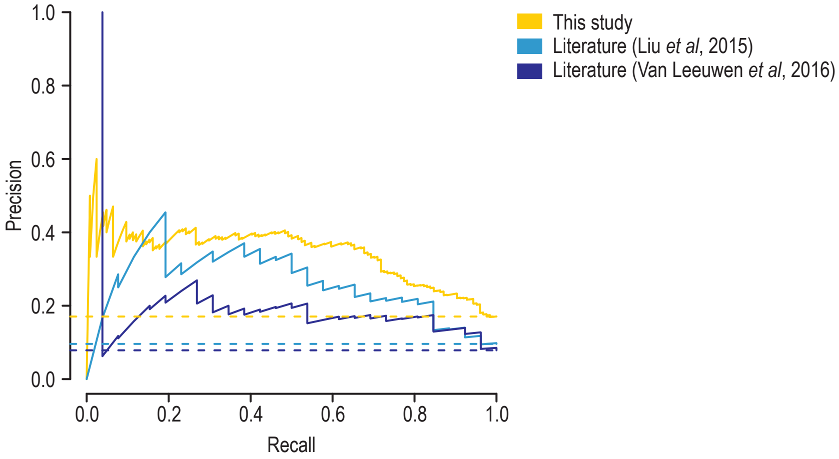

Fig. S7. Dispensable essential gene prediction.
Precision (fraction of true positives among all positives) versus recall (number of true positives) curves for a dispensable gene prediction model based on a random forest algorithm. True positive dispensable genes were defined by either excluding a subset of dispensable genes found in this study from the training set ("this study"), or by using dispensable essential gene sets identified in other studies but not tested in our experiment (Liu *et al*, 2015; Van Leeuwen *et al*, 2016).

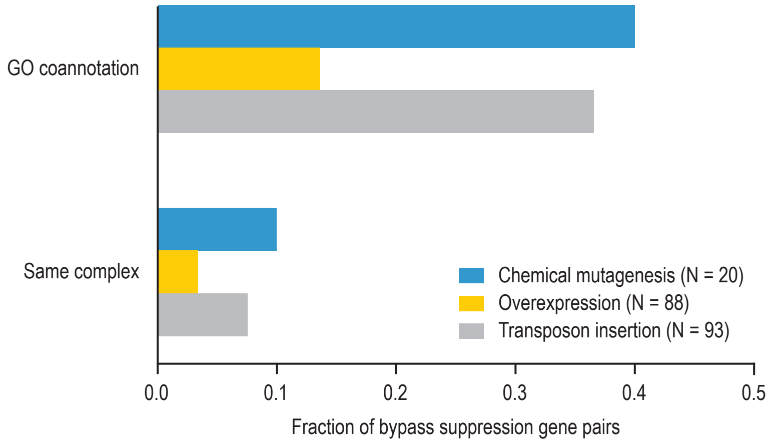

Fig. S8. Bypass suppressors identified in *S. pombe*.
Fraction of gene pairs involved in a bypass suppression interaction in the fission yeast *S. pombe* (Li *et al*, 2019) that share a GO coannotation or encode members of the same protein complex. Suppression interactions were grouped by the method of suppressor isolation: either chemically induced genomic mutations, high overexpression, or random transposon insertions. The latter category involves both activation and deletion events.
